# Vertical Microcavity Organic Light-emitting Field-effect Transistors

**DOI:** 10.1038/srep23210

**Published:** 2016-03-17

**Authors:** Yongsheng Hu, Jie Lin, Li Song, Qipeng Lu, Wanbin Zhu, Xingyuan Liu

**Affiliations:** 1 State Key Laboratory of Luminescence and Applications, Changchun Institute of Optics, Fine Mechanics and Physics, Chinese Academy of Sciences, Changchun 130033, China; 2 State Key Laboratory of Applied Optics, Chinese Academy of Sciences, Changchun 130033, China

## Abstract

Organic light-emitting field-effect transistors (OLEFETs) are regarded as a novel kind of device architecture for fulfilling electrical-pumped organic lasers. However, the realization of OLEFETs with high external quantum efficiency (EQE) and high brightness simultaneously is still a tough task. Moreover, the design of the resonator structure in LED is far from satisfactory. Here, OLEFETs with EQE of 1.5% at the brightness of 2600 cdm^−2^, and the corresponding ON/OFF ratio and current efficiency reaches above 10^4^ and 3.1 cdA^−1^, respectively, were achieved by introducing 1,4,5,8,9,12-hexaazatriphenylene-hexacarbonitrile (HAT-CN) as a charge generation layer. Moreover, a vertical microcavity based on distributed Bragg reflector (DBR) and Ag source/drain electrodes is successfully introduced into the high performance OLEFETs, which results in electroluminescent spectrum linewidth narrowing from 96 nm to 6.9 nm. The results manifest the superiority of the vertical microcavity as an optical resonator in OLEFETs, which sheds some light on achieving the electrically pumped organic lasers.

Organic light-emitting field-effect transistors (OLEFETs) are a novel kind of electroluminescent (EL) devices with field-effect characteristic, which show great potential for use in low-cost displays and integrated optical communications^1^,^2^. The unique architecture of OLEFETs also enables them with minimum excitonic losses, efficient light confinement and high current density, thus is conducive to the realization of net gain in laser devices. Therefore, OLEFETs are also regarded as a promising device architecture for fulfilling electrical-pumped organic lasers^3^,^4^,^5^.

To demonstrate electrically pumped lasing in OLEFETs, one challenge is how to build a suitable resonator which could provide effective optical feedback in OLEFETs. More specifically, obtaining a narrow band emission spectrum by incorporating an optical resonator while maintaining high device performance (i.e., high emission efficiency and high brightness) is an important step towards electrically pumped organic lasers based on the architecture of OLEFETs. Resonator structures such as distributed feedback (DFB) cavity^6^, single crystal based F-P cavity^7^ as well as grating cavity^8^ have been investigated in OLEFETs, however, the linewidth (full width at half maximum, FWHM) was limited in the range of 10~25 nm. There were some other reports on 1D/2D photonic crystal imported in OLEFETs which resulted in very narrow linewidth, however, the fabrication process of photonic crystals, such as electron-beam lithography, reactive ion etching and lamination, would probably bring some physical damage to the morphology of OLEFETs, leading to poor device performance^9^,^10^. Considering that the emission from OLEFETs is mainly in the vertical direction, it would be more appropriate to introduce a resonator vertically. Recently, OLEFETs with large emission area under the source/drain (S/D) electrodes have been reported by several groups^11^,^12^,^13^,^14^. Although the absorption loss from the metal electrodes would increase, it is of great convenience to build a vertical microcavity in such an OLEFET, since the metal electrodes can act as one of the mirrors of the cavity. Moreover, since distributed Bragg reflector (DBR) is uaually used as the bottom mirror for a vertical microcavity, its relatively smooth morphorlogy is beneficial for the growth of high quality organic films on top, which would be a big obstacle for the optical feedback structures such as DFB and 1D/2D photonic crystal due to their large morphorlogy fluctuations. Very recently, we have reported a DBR/Au vertical microcavity in an area-emitting OLEFET where the peak of the EL spectrum could be adjusted^15^. However, the FWHM of the EL spectrum was as large as 26 nm due to the low reflectance of the Au mirror since a thin Au film has to be used in order to get enough emission intensity. How to build a vertical microcavity in OLEFETs to obtain narrower EL spectrum is one of the questions that we concern in this work.

On the other hand, it is generally believed that heterostructured OLEFETs consist of a p-type and (or) a n-type carrier transport layer and an emissive layer (EML) have the potential in achieving better overall performance compared with single layer devices since high mobility materials and high EL efficiency materials can be simultanously introduced as the carrier transportation layer (CTL) and EML, respectively^16^,^17^,^18^. However, there are usually large carrier injection barriers both at the interfaces of electrodes/CTL and CTL/EML, which would hinder the injection or transportation of one or two kinds of the carriers. Large amount of studies have been focused on the interfaces of electrode/CTL and insulator/CTL^19^,^20^, while few reports on the interface between the organic layers.

Here, we used 1,4,5,8,9,12-hexaazatriphenylene-hexacarbonitrile (HAT-CN), a strong electron acceptor with high electron affinity^21^,^22^, to act as a charge generation layer between the hole transportation layer and hole buffer layer of heterostructured OLEFETs, where high EQE and high brightness were obtained simultaneously. More importantly, low-cost and stable metal Ag was employed as the S/D electrodes, despite that the electrodes with both high work function (i.e., Au) and low work function (i.e., Ca, Mg) are required for high performance OLEFETs^19^,^23^. Compared to other metals, Ag is the most widely used metal mirror in microcavity since it can provide higher reflectance in the visible spectral region. We successfully built a DBR/Ag microcavity in the OLEFETs, which shows strong microcavity effect. By optimizing the cavity length and the reflectance of the top mirror, FWHM as narrow as 6.9 nm was obtained. The results manifest the superiority of vertical microcavity as an optical resonator for potential application in electrically pumped lasing techniques in OLEFETs.

## Results

**Figure 1:**
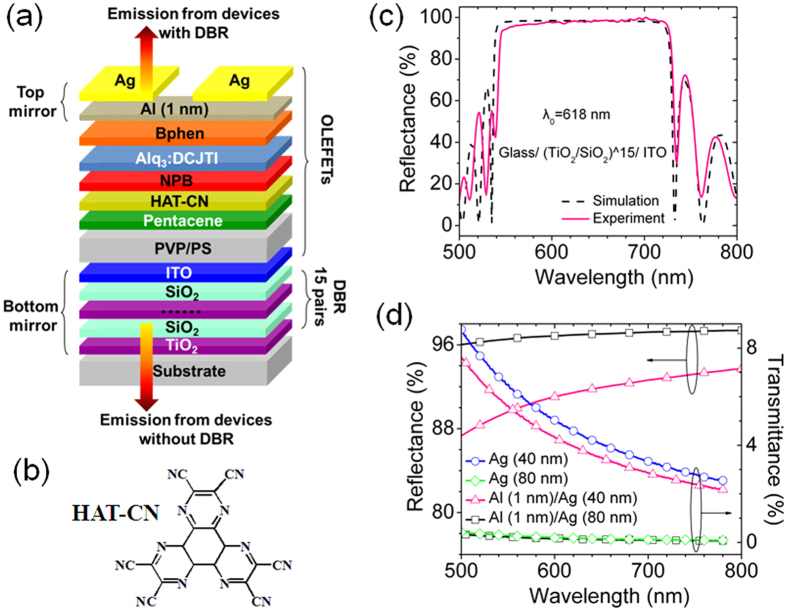
Device structure and optical properties of mirrors. (**a**) Schematic of a microcavity OLEFET. (**b**) Chemical structure of HAT-CN. (**c**) Simulated and experimental reflectance of the bottom mirror. (**d**) Reflectance and transmittance of the top mirror for 40 nm and 80 nm Ag; the transmittance of 40 nm and 80 nm Ag without Al.

Figure 1a shows the schematic of the device: the bottom mirror was formed by DBR and a thin layer of ITO. The DBR was fabricated by depositing 15 pairs of TiO_2_/SiO_2_ while the ITO also functioned as the gate electrode. Poly-4-vinylphenol (PVP) /polystyrene (PS) acted as the dielectric. Pentacene and HAT-CN (Fig. 1b) were used as the hole transportation layer and charge generation layer, respectively. N,N′-di(naphthalene-1-yl)-N,N′-diphenyl-benzidine (NPB) was selected as a hole buffer layer since its highest occupied molecular orbital (HOMO) level lies between that of pentacene and the EML. The EML used was a typical material in organic light-emitting diodes (OLEDs): tris(8-hydroxyquinoline)-aluminum(III) (Alq_3_) : 2-[2-(1-Methylethyl)-6-[2-(2,3,6,7-tetrahydro-1,1,7,7-tetramethyl-1H,5H-benzo[ij]quinolizin-9-yl)ethenyl]-4H-pyran-4-ylidene]propanedinitrile (DCJTI). Bathophenanthroline (Bphen) was used both as the electron-injecting layer and hole-blocking layer due to its low lowest unoccupied molecular orbital (LUMO) level and high HOMO level, respectively. Ag was used as the S/D electrodes, before which an ultra thin layer of Al (1 nm) was inserted to further promote the electron injection as demonstrated in top-emitting OLEDs^24^,^25^. Figure S1 shows the molecular structures of the active materials and the energy schematic diagram of the devices. The thin layer of Al together with the Ag layer formed the top mirror. Fig. 1c,d shows the reflectance of the bottom mirror and top mirror, respectively. The reflectance of the bottom mirror is about 97.9% around the central wavelength of the bottom mirror (618 nm), which is a little lower than the simulated result (98.4%). For the top mirror with 80 nm and 40 nm Ag, the reflectance is about 96.9% and 91.4%, respectively, at around 618 nm. Since the reflectance of the top mirror is lower than that of the bottom mirror, the light is collected from the top of the devices with DBR while from the bottom of the devices without DBR. Figure 1d also shows the transmittance of 80 nm and 40 nm Ag both with and without Al. The transmittance of Ag is slightly lowered when Al is inserted. Considering the large absorption of Al in the visible spectral region, it is reasonable to have a thin thickness of Al.

**Figure 2:**
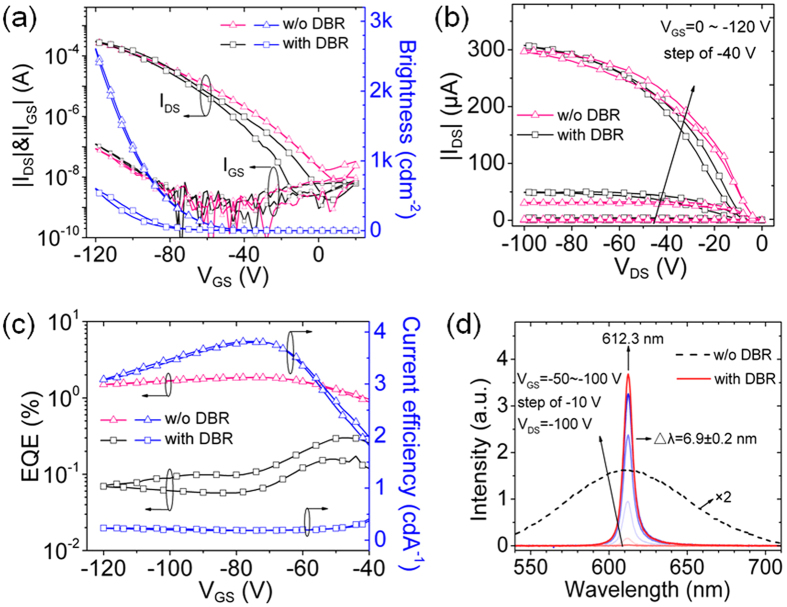
Characteristics of devices. (**a**) Transfer characteristic (V_DS_ = −100 V) and the corresponding brightness and gate leakage current, (**b**) output characteristic, (**c**) the corresponding EQE and current efficiency (V_GS_ = −40~ −120 V) for devices with and without DBR. (**d**) EL spectra for devices with (V_DS_ = −100 V, V_GS_ changes from −50 V to −100 V in step of −10 V) and without DBR (V_DS_ = V_GS_ = −100 V, the intensity is twice magnified).

Figure 2 presents the main optoelectrical characteristics for devices with and without DBR. The thickness of Ag is 80 nm for both of the devices. Figure 2a is the transfer characteristic (V_DS_ = −100 V) and the corresponding brightness along with the gate leakage current (I_GS_). The transfer characteristic for both of the devices is almost the same, which indicates that the DBR does not influence the film quality of the top organic layers. Both of them exhibit p-type transportation characteristic, which is due to the high hole mobility of pentacene and the introduction of the charge generation layer. The hole mobility and ON/OFF ratio are 0.54 ± 0.2 cm^2^V^−1^s^−1^ and above 10^4^, respectively. The brightness increases with the increasing drain current (I_DS_) for both of the devices, which is consistent with those of heterostructured unipolar OLEFETs^18^,^26^. The maximum brightness for the device without DBR reaches 2600 cdm^−2^, which is among the best results ever reported for red OLEFETs. In contrast, the brightness for the device with DBR is only 600 cdm^−2^, which is mainly resulted from the top emission mode, since the transmittance of the top mirror is very low. Not surprisingly, when biased at the same condition, the brightness for device without DBR from the top is only 435 cdm^−2^ (Figure S2 and Table 1). The gate leakage currents remain at a relatively low level of about 10^−7^ A, which is about 3 orders of magnitude lower than the drain currents, thus their influence for the performance of the devices can be excluded.

Figure 2b is the output characteristic for both of the devices, where typical unipolar transportation characteristic with distinct linear and saturation regimes can be found. Figure 2c shows the corresponding EQE and current efficiency. Considering that the spatial distribution of emission intensity for a microcavity device is no longer Lambertian, the EQE in this work was calculated based on the actual spatial distribution of emission intensity of the devices. The EQE for the device without DBR reaches as high as 1.8% around V_GS_ = −70 V and maintains at 1.5% at the brightness of 2600 cdm^−2^. The high EQE mainly results from the high exciton recombination efficiency, which is typically in the order of 10^−2^~10^0^% in the literatures^14^,^19^,^23^, due to the well energy level alignment between the organic layers. Given to the formular^27^ that Φ_EQE_ = Φ_out_ × Φ_spin_ × Φ_PL_ × Φ_rad_, where Φ_out_ is the outcoupling efficiency, Φ_spin_ is a factor to take account of spin statistics, Φ_PL_ is the fluorescent quantum efficiency that is ~68% by the measurement, and Φ_rad_ is the exciton recombination efficiency. When assuming Φ_out_ and Φ_spin_ as 20% and 25%, respectively, the Φ_rad_ can be as high as 44% for EQE of 1.5%. As for device with DBR, the maximum EQE is about 0.3% around V_GS_ = −50 V and reduces to 0.07% when the brightness gets the maximum. The current efficiency for the device without DBR is more than 3.1 cdA^−1^, which is even comparable with typical OLEDs using Alq_3_:DCJTI as the EML^28^. The current efficiency for the device with DBR is 0.2 cdA^−1^. The lower EQE as well as current efficiency for the device with DBR results also from the low transmittance of the top mirror. Figure 2d shows the EL spectra for both of the devices. The peak of the spectrum of the device with DBR is 612.3 nm, which is similar to that of the device without DBR and is near the central wavelength of the bottom mirror. This attributes to the accurate adjustment of the cavity length. The EL spectrum for the device with DBR keeps unchanged with the increasing of V_GS_ from −50 V to −100 V. The FWHM of the spectrum for the device with DBR is only 6.9 nm, while that for device without DBR is 96 nm. The narrow FWHM mainly results from the high reflectance of the two mirrors of the microcavity, which will be discussed in detail later. Table 2 compares the reported results for OLEFETs with different types of resonators. The microcavity in this work shows a better potential to reduce the FWHM while maintaining relatively high EQE and brightness.

Despite of the different emission directions between the two devices, the inferiority of the emission characteristics (brightness, EQE, and current efficiency) for the device with DBR seems quite puzzled since the microcavity is generally used in OLEDs to improve the EL performance^29^. In order to get a high EL efficiency, the reflectance of the mirror at the side of light output in normal microcavity OLEDs should be at a lower level so that a weak microcavity effect can be formed to allow more optical modes to couple out together. While, the mirror with quite high reflectance was used here so that only limited or single resonant mode can couple output, resulting in a narrowed linewidth. When a high reflectance mirror is used in microcavity OLEDs, the EL intensity would be much lower than that without microcavity^30^. In other words, the decrease of the brightness, EQE and current efficiency for the device with DBR compared to that of the device without DBR indicates a lower integrated intensity for the microcavity. However, the emission from the resonance optical mode has been enhanced significantly. Given from the EL spectra in Fig. 2d, the intensity at the resonance wavelength (612.3 nm) for the device with DBR is significantly enhanced, which is 4.5 times of that for device without DBR. Theoretically, the enhancement of the emission intensity along the cavity axis at the resonance wavelength is given by^31^,^32^:


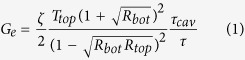


where, *R*_*bot*_ and *R*_*top*_ are the reflectance of the bottom mirror and the top mirror, respectively; *T*_*top*_ is the transmittance of the top mirror and *ζ* is the antinode enhancement factor. *ζ* has a maximum value of 2 when the emitting dipoles (i.e., the exciton recombination zone) is located exactly at the antinode of the standing wave. *τ*_*cav *_/*τ* is the ratio of exciton lifetime in the cavity device and the noncavity device. For device with DBR in this work, *R*_*bot*_, *R*_*top*_ and *T*_*top*_ at 612.3 nm are 97.9%, 96.9%, and 0.15%, respectively (Fig. 1). Considering that the thickness of the EML is 28 nm, the emitting dipoles can not locate exactly at the antinode of the standing wave, here, we take *ζ* = 1.4^31^. Meanwhile, the exciton lifetime is likely to be shorter due to cavity effects, we take *τ*_*cav *_/*τ* = 0.8^32^, then we can calculate that *G*_*e*_ = 5, which agrees quite well with the measured value of 4.5.

**Figure 3:**
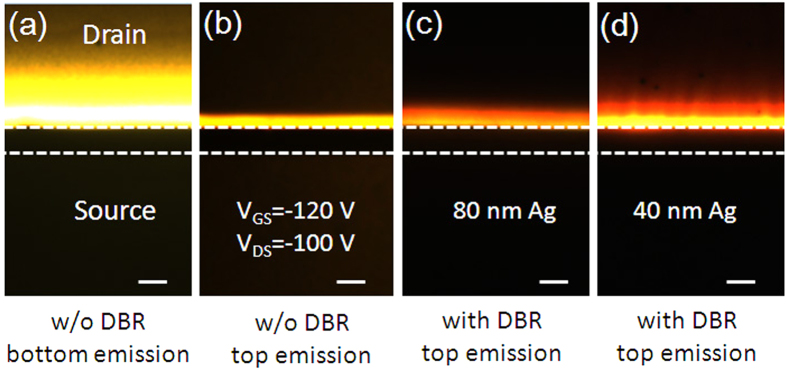
Optical images of devices. Optical images for the device without DBR, (**a**) bottom emission, (**b**) top emission. Optical images for the DBR device with different top mirrors, (**c**) 80 nm Ag, (**d**) 40 nm Ag. All the devices are biased at V_GS_ = −120 V, V_DS_ = −100 V. The scale bar is 50 μm.

Figure 3 shows the optical images for different devices biased at V_GS_ = −120 V, V_DS_ = −100 V. All of the devices exhibit area-emitting characteristic under the drain electrode. The width of the emission area for the device without DBR (Fig. 3a) is about 100 μm, while is only 20 μm when observed from the top (Fig. 3b), which is due to the low transmittance of the Ag electrode. As for the devices with DBR, the width of the emission area is about 33 μm when the thickness of Ag is 80 nm (Fig. 3c), and it extends to about 45 μm when the thickness of Ag reduces to 40 nm (Fig. 3d). The dynamic behavior of the emission area when V_GS_ sweeps can be found in Videos S1 and S2. The maximum intensity of the emission is near the edge of the drain electrode, and the area extends outward as V_GS_ increases. It indicates that large amount of holes are accumulated in the hole transportation layer under the drain electrode^17^.

Figure S3a shows the UV-vis-NIR absorption spectra of a pentacene film, a HAT-CN film and a HAT-CN-doped pentacene film (50% mol HAT-CN). The newly emerged charge transfer peak around 950 nm in the HAT-CN-doped pentacene film indicates that there is charge transfer between HAT-CN and pentacene^33^, which is probably the main reason for the high performance of the OLEFTEs. For comparison, the electrical transfer and luminescence characteristics for device (80 nm Ag) without HAT-CN layer and DBR are presented in Figure S4. The maximum drain current is dramatically lowered by two orders of magnitude to the range of a few microampere. The brightness, EQE, and current efficiency are also reduced to a few cdm^−2^, 0.4% and 0.5 cdA^−1^, respectively. Sun, *et al.* investigated the behavior of carrier transportation when HAT-CN contacts with different types of p-type organic semiconductors^22^. They found that when under a certain bias, the electron could tunnel, which follows the Zener tunneling rule, from the HOMO level of p-type materials into the LUMO level of HAT-CN, thus, generating an electron in the LUMO level of HAT-CN and producing a hole in the HOMO level of p-type materials. In our case, when the devices are operating in the hole accumulation mode, assisted by the strong electric field near the source electrode, extra holes can be generated in the HOMO level of pentacene resulted from the electron tunneling from the HOMO level of pentacene into the LUMO level of HAT-CN. The generated holes together with the holes injected from the source electrode, will accumulate at the interface between pentacene and the insulate layer due to the field effect and transport towards the drain electrode. The holes will then accumulate under the drain electrode and inject to the HOMO level of the EML. The use of NPB can make the process of injection more efficient since the hole injection barrier is lowered. The holes will recombine with the electrons injected from the drain electrode in the LUMO level of the EML, which results in the intense emission beneath the drain electrode. The high HOMO level of Bphen can block the holes which further promotes the exciton recombination efficiency. The schematic representation of the carrier injection and transportation is shown in Figure S3b.

**Figure 4:**
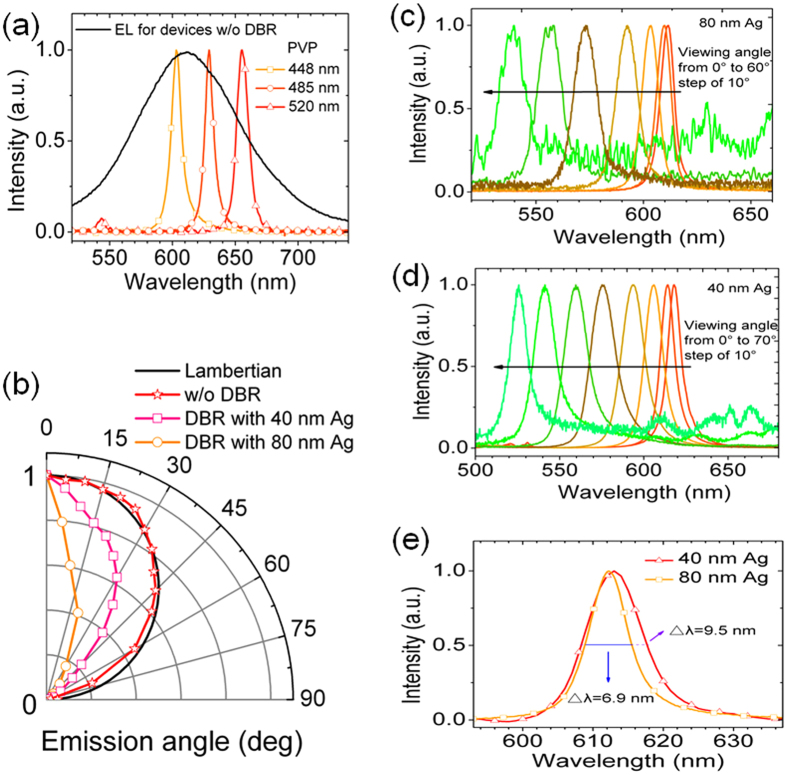
EL emission characteristics of devices. (**a**) EL spectra for DBR devices with different thickness of PVP. (**b**) Spatial distribution of emission intensity for different devices. Normalized EL spectra under different viewing angles for DBR devices with (**c**) 80 nm Ag, (**d**) 40 nm Ag. (**e**) Normalized EL spectra for DBR devices with different thickness of Ag.

Next, we will focus on the characteristics related to the microcavity. In a microcavity, only the optical standing waves can be existed, which means that the emission wavelengths have to meet the resonance condition^34^:


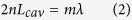


where, *L*_*cav*_ and *n* are the effective cavity length and the effective refractive index, respectively, m is an integer (in our case, *m* = 3). In the device with DBR that we discussed above, the thickness of PVP (460 nm) is carefully optimized so that the resonance wavelength of the microcavity can agree well with the emission peak of the EML material in the free space, thus a maximum emission intensity can be obtained. Figure 4a shows the EL spectra for DBR devices with different thicknesses of PVP. As the thickness of PVP increases, a red shift of the EL spectrum is observed. When the EL peak shifts to 655 nm, a second peak appears at 545 nm, which results from the resonance optical mode out of the stop-band of the microcavity. Figure 4b shows the spatial distribution of emission intensity for different devices. As expected, the spatial distribution for the device without DBR is similar to that of Lambertian. As for devices with DBR, when the thickness of Ag is 40 nm, 80% of the emission intensity is concentrated within 45° from the normal direction. When the thickness of Ag increases to 80 nm, the emission intensity is further concentrated within 30° from the normal direction. This indicates that the microcavity has intense influence on the spatial distribution of emission intensity: the higher the reflectance of the mirror is, the more the emission intensity will be concentrated to the cavity axis. Another characteristic for a microcavity device is that the EL spectrum is viewing angle depended^31^. Figure 4c,d shows the EL spectra under different viewing angles for DBR devices with 80 nm Ag and 40 nm Ag, respectively. As the viewing angle increases, a significant blue shift of the EL spectrum is observed. When the viewing angle increases to 60°, the EL peaks in both of the devices shift to about 540 nm, which means that the blue shift is more than 70 nm.

Quality factor (Q factor), which is determined by the total energy storages in a cavity and the energy dissipation rate, is an important parameter to characterize an optical cavity^35^. High Q factor is prefered since the threshold of microcavity laser devices can be significantly reduced, which means that it is much easier to achieve lasing^34^. The Q factor is given in the following equation^35^,^36^:


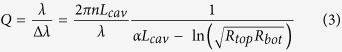


where *L*_*cav*_ and *n* are the effective cavity length and the effective refractive index, respectively, *α* is an average distributed loss constant. The most common loss mechanism in optical cavity includes absorption and scattering of the mirrors and the inner components as well as the diffraction loss^35^. Given to Equation 3, supposing that *α* is a constant, then a longer cavity length and a higher reflectance will result in a higher Q factor and a narrower spectrum linewidth. Given to equation (2), the cavity length for the microcavity OLEFETs in this work is 3λ/2n, while it is usually λ/2n for microcavity OLEDs. The cavity length for microcavity OLEDs is restricted by the high resistivity of the organic layers since the current will decrease significantly as the thickness of the organic layers increases. Hoffmann, *et al.*^37^ investigated microcavity OLEDs with different cavitylength, i.e., λ/2n, λ/n and 3λ/2n, which verified that both the current and the EQE of the devices decreased as the cavity length increased. Therefore, benefiting from the wide adjustment of the thickness of the insulator, the microcavity OLEFETs are able to achieve higher Q factor than common microcavity OLEDs. Figure 4e shows the EL spectra for DBR devices with different thickness of Ag. The linewidth of the EL spectrum for the DBR device with 40 nm Ag extends significantly from 6.9 nm to 9.5 nm and the Q factor decreases from 89 to 64 compared with that of DBR device with 80 nm Ag for its lower reflectance of the top mirror. However, since the transmittance is higher for 40 nm Ag, the EL emission intensity is much higher than that of the DBR device with 80 nm Ag. Figure S2 gives the transfer characteristic and the corresponding brightness, gate leakage current, EQE, and current efficiency for the DBR device with 40 nm Ag.

From Equation 3, we can also see that a higher Q factor and narrower linewidth can be achieved by minimizing *α*. This reminds us that a high reflectance mirror with lower absorption loss, i.e., hybrid mirror composed of thin Ag and dielectric cap layers, or dielectric-only mirror^34^,^38^, should be a much better choice for the microcavity mirror. It is ideal to combine a dielectric-only mirror with an OLEFET that emitting in the channel since the absorption loss from the metal electrode could also be minimized. To move the emission area away from the electrode, possible methods include using asymmetrical S/D electrodes, doping the electron injecting layer, and introducing materials with high electron mobility^16^,^19^. We believe that with further optimization of microcavity structures and the fabrication process, vertical microcavity OLEFETs with higher Q factor and better emission characteristics could be achieved.

In conclusion, we have demonstrated high performance OLEFETs by incorporating a novel charge generation layer of HAT-CN. The devices have shown EQE of 1.5% at the brightness of 2600 cdm^−2^, and the corresponding ON/OFF ratio and current efficiency reaches above 10^4^ and 3.1 cdA^−1^, respectively, which is among the best results ever reported. Owing to the area-emitting characteristic and the Ag S/D electrodes, an effective DBR/Ag vertical microcavity structure is introduced into the high performance OLEFETs. The influences of the cavity length and the reflectance of the top mirror have been investigated and EL spectrum linewidth as narrow as 6.9 nm has been obtained. Although further optimization of the microcavity structure and improvement of the fabrication process are needed to overcome the limited Q factor, our work manifests the superiority of the vertical microcavity as an optical resonator for the combination with OLEFETs, which provides a useful reference for designing and achieving electrical-pumped organic lasers.

## Methods

### Device Fabrication

TiO_2_ (66 nm), SiO_2_ (106 nm), and ITO (78 nm) were deposited onto the glass substrate by E-beam evaporation under a base pressure of 1.5 × 10^−4^ Pa, evaporation rate of 2 Å/s, and substrate temperature of 300 °C. An end-Hall ion source was used to assist the deposition. PVP (460 nm) and PS (30 nm) were spun coated in the way reported elsewhere^39^. Pentacene (12 nm), HAT-CN (2 nm), NPB (10 nm), Alq_3_:DCJTI (28 nm, weight concentration of 1.5%), Bphen (14 nm) and Al (1 nm) were successively thermal evaporated with the rate of 0.2, 0.1, 0.2, 2, 0.2 and 0.2 Å/s, respectively. Ag was evaporated through a shadow mask with channel length and width of 45 μm and 3000 μm, respectively. The devices were encapsulated with UV glue in the glovebox (H_2_O, O_2_ < 0.1 ppm) before testing.

### Device Characterization

The electrical characteristics were performed by Keithley 4200 SCS at room temperature under air ambient. The photocurrent was recorded by HAMAMATSU S1336 photodiode. The optical images were captured by Olympus BX51TRF CCD microscope. The EL spectra were measured by AvaSpec-ULS2048L fiber spectrometer. The absorption spectra were recorded by Shimadzu UV-3101PC UV-vis-NIR spectrophotometer. The reflectance and transmittance were recorded by PerkinElmer Lambda 1050 UV-vis-NIR spectrophotometer. Absolute fluorescent quantum yield measurements were performed with a calibrated integrating sphere on an Edinburgh FLS920 spectrometer. The carrier mobilities were calculated by the formula for the saturation regime: I_DS_ = μC_i_(W/2L)(V_GS_–V_T_)^2^, (where μ is the field-effect mobility, C_i_ is the gate dielectric capacitance density, V_T_ is the threshold voltage, W and L are the channel width and length, respectively). The brightness was calculated by comparing the photocurrent with a standard OLED of known brightness (1000 cdm^−2^) and emission area (3 mm × 1 mm) with structure of ITO/NPB/Alq_3_:DCJTI/Alq_3_/LiF/Al. The EQE was calculated from the brightness, the drain current, the EL emission spectrum, and the spatial distribution of emission intensity of the devices.

## Additional Information

**How to cite this article**: Hu, Y. *et al.* Vertical Microcavity Organic Light-emitting Field-effect Transistors. *Sci. Rep.* **6**, 23210; doi: 10.1038/srep23210 (2016).

## Supplementary Material

Supplementary Information

Supplementary Video 1

Supplementary Video 2

## Figures and Tables

**Table 1:** Comparison of the characteristics for different devices.

Device	w/o DBR 80 nm Ag (bottom emission)	w/o DBR 80 nm Ag (top emission)	with DBR 80 nm Ag (top emission)	with DBR 40 nm Ag (top emission)
Max. Brightness (cdm^−2^)	2600	435	600	1900
EQE at Max. Brightness (%)	1.5	0.05	0.07	0.39
Current efficiency (cdA^−1^)	3.1	0.1	0.2	1
Linewidth (nm)	96	96	6.9 ± 0.2	9.5 ± 0.2
Width of emission area (μm)	100 ± 5	20 ± 2	33 ± 2	45 ± 2
Direction (80% energy coverage)	70°	70°	45°	30°
Q factor	/	/	89	64

**Table 2:** Comparison of OLEFETs with vertical microcavity and other reported resonators.

Ref.	Device Architecture	Resonator Type	FWHM w/o resonator (nm)	FWHM with resonator (nm)	EQE (%)	Brightness (cdm^−2^)
6	Single layer Polymer	DFB	~80	~27	N/A	N/A
7	Single Crystal	F-P	~36	~14	N/A	N/A
8	Single Crystal	DFB	~58	~26	1.72 × 10^−3^	N/A
9	Single Crystal	2D DFB	N/A	9.8@main peak	N/A	N/A
10	Single Crystal	DFB	42.1	2.05	1.72 × 10^−5^	N/A
This work	Multilayer Small Molecule	Microcavity	96	6.9	0.07	600

The EQE and brightness are values with resonators.
