# Supplementary material for: Vertical Microcavity Organic Light-emitting Field-effect Transistors
Source: Sci Rep. 2016 Mar 17;6:23210. doi: 10.1038/srep23210 (PMC4794712; doi:10.1038/srep23210)
Supplement: Supplementary Information [file srep23210-s1.doc]

Supporting Information

**Vertical Microcavity Organic Light-emitting Field-effect Transistors**

Yongsheng Hu^1^, Jie Lin^1,*^, Li Song^1^, Qipeng Lu^2^, Wanbin Zhu^2^, and Xingyuan Liu^1,*^

^1^State Key Laboratory of Luminescence and Applications, Changchun Institute of Optics, Fine Mechanics and Physics, Chinese Academy of Sciences, Changchun 130033, China
^2^State Key Laboratory of Applied Optics, Chinese Academy of Sciences, Changchun 130033, China

*Email: linj@ciomp.ac.cn; liuxy@ciomp.ac.cn


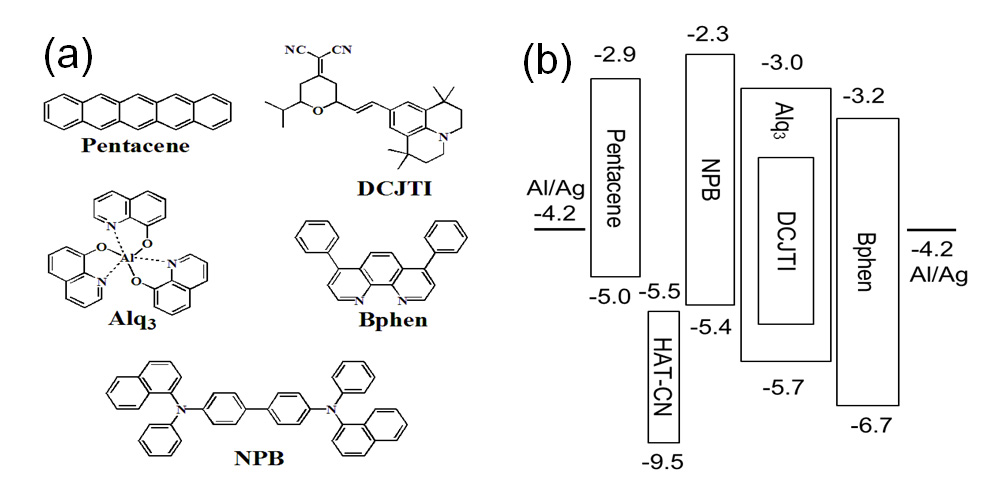


**Figure S1** (a) Molecular structures of the active materials. (b) Energy schematic diagram of the devices.


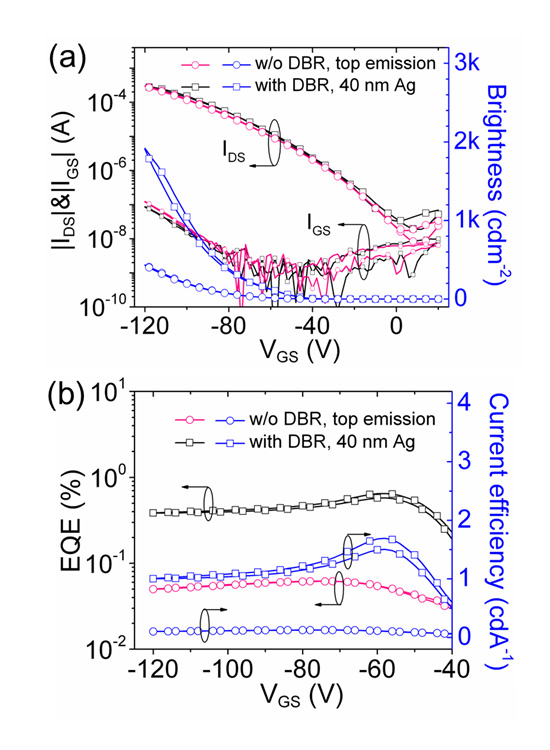


**Figure S2** (a) Transfer characteristic (V_DS_= −100 V) and the corresponding brightness and gate leakage current. (b) The corresponding EQE and current efficiency (V_GS_= −40~ −120 V) for devices with DBR (40 nm Ag) and without DBR (top emission).


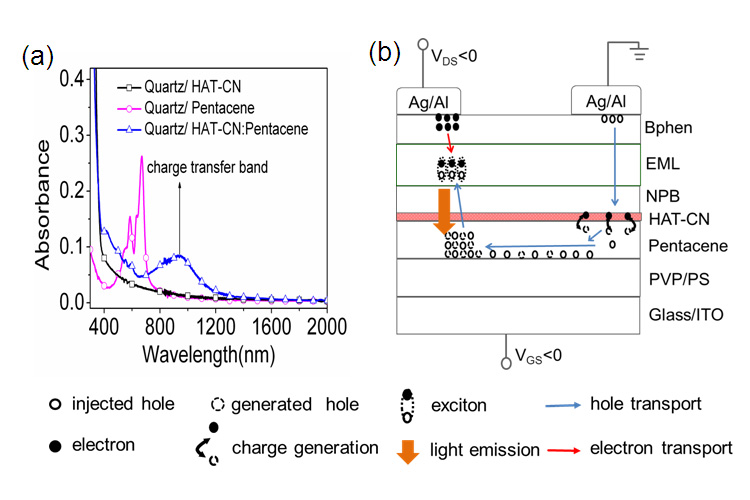


**Figure S3.** Absorption spectra of films and operation mechanism of device. (a) UV-vis-NIR absorption spectra of a pentacene ﬁlm (60 nm), a HAT-CN ﬁlm (60 nm), and a HAT-CN-doped pentacene ﬁlm (60 nm, 50% mol HAT-CN), (b) schematic representation of the carrier injection and transportation.


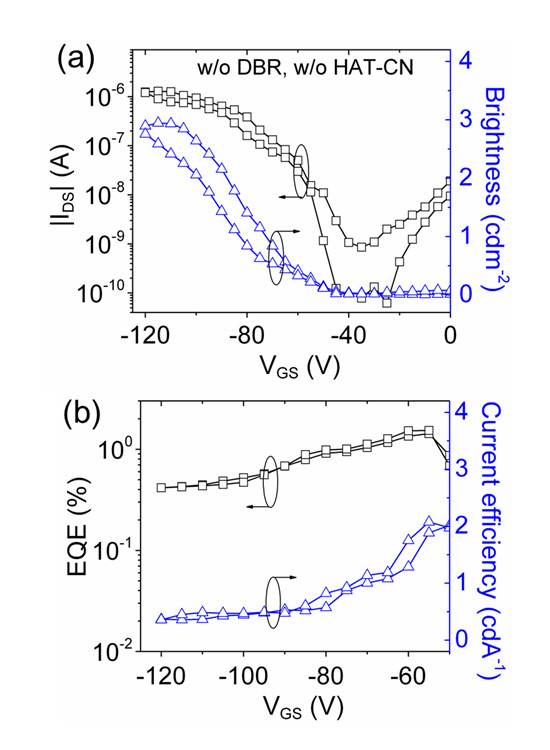


**Figure S4** (a) Transfer characteristic (V_DS_= −100 V) and the corresponding brightness, (b) EQE and current efficiency (V_GS_= −50~ −120 V) for devices without DBR and HAT-CN layer.

**Video S1** The dynamic behavior of the emission area when V_GS_ sweeps from 20 V to −120 V (V_DS_= −100 V) for device without DBR (bottom emission, 20× objective lens), the drain electrode is at the upside of the screen.

**Video S2** The dynamic behavior of the emission area when V_GS_ sweeps from 20 V to −120 V (V_DS_= −100 V) for device with DBR (80 nm Ag, 10× objective lens), the drain electrode is at the downside of the screen.
